# The COVID-19 Pandemic Seen from a Syndemic Perspective: The LGBTQIA2SP+ Community

**DOI:** 10.3390/idr13040078

**Published:** 2021-10-03

**Authors:** Nicola Luigi Bragazzi

**Affiliations:** Laboratory for Industrial and Applied Mathematics (LIAM), Department of Mathematics and Statistics, Faculty of Science, York University, Toronto, ON M3J 1P3, Canada; robertobragazzi@gmail.com

An adverse condition or a disease can (either directly or indirectly) interact in a synergistic fashion with other adverse conditions or diseases/maladies, and co-cluster together with them: this fundamental observation is at the basis of the term “syndemic” (a portmanteau for “synergistic epidemic”). This term was introduced for the first time by the American medical anthropologist Merrill Singer (McKeesport, Pennsylvania, USA, 1950), who developed the conceptual framework of the so-called “syndemics theory” in the mid-1990s [1]. Singer noted that women from ethnic minorities reported multiple comorbidities and co-occurring conditions, such as human immunodeficiency virus (HIV)/acquired immunodeficiency syndrome (AIDS), violence, substance abuse, and poverty/marginalization: this overlap resulted in a particularly worse health outcome, with each of the conditions magnifying the negative effects of the other in an additive way [1].

The syndemics theory has enabled scholars to identify particular sub-populations and specific community strata at higher risks for developing/spreading HIV/AIDS, such as gay and bisexual individuals and other men having sex with men (GBMSM), or youth living with HIV (YLWH). Other examples of vulnerable and frail populations include individuals suffering from mental health disorders and other issues (such as depression, binge drinking, (poly-)substance use, street drug use, psychological distress, childhood sexual abuse, intimate partner violence and sexual assault as well as other psychosocial risk factors) or subjects experiencing stigma, a lack of strong social support networks, other psychological burdens, and/or low socio-economic status [1,2,3,4,5,6,7,8].

Adopting a bio-psycho-social perspective and, more specifically, a syndemic lens is of paramount importance in devising and designing public health preventative strategies and interventional approaches aimed at improving and enhancing community wellbeing, which results from the complex, non-linear interaction between societal, economic–financial, environmental, cultural, and political variables [8].

In late December 2019, an emerging coronavirus, initially named “2019 novel coronavirus” (2019-nCoV) and subsequently termed “Severe Acute Respiratory Syndrome-related Coronavirus type 2” (SARS-CoV-2), was detected in the metropolitan city of Wuhan, province of Hubei, mainland China. It was identified as the infectious agent responsible for the still-ongoing “2019-nCoV acute respiratory disease” or “Coronavirus Disease 2019” (COVID-19) pandemic. Since then, the virus has spread globally and has caused a dramatic toll of infections (more than 230 million cases) and more than 4.7 million deaths, affecting 221 countries and territories around the world.

Some authors have proposed terming the still-ongoing COVID-19 pandemic a syndemic [9], showing that the virus, by interacting with underlying societal iniquities, communicable and non-communicable diseases, and effects generated by climate change, has amplified already-existing distortions, resulting in an excess of mortality and morbidity.

Due to the emerging nature of the pathogen and with populations being largely immunologically naïve to the infectious agent, given the lack of available effective drugs or vaccines, public health authorities had to implement non-pharmaceutical interventions (NPIs), such as enhanced hygiene practices (including the use of face masks), social/physical distancing, self-isolation, quarantine and even lockdowns of entire territories/cities, later extended at the entire national level. On the one hand, these measures have succeeded in curbing the transmission of the virus and in flattening the epidemic curve. On the other hand, these public health interventions and the syndemic nature of the viral agent have contributed to generating a relevant economic and psychological burden.

The LGBTQIA2SP+ (Lesbian, Gay, Bisexual, Trans, Queer/Questioning, Intersex, Asexual/Aromantic and Allied, Two-Spirited and Polysexual/Pansexual) communities have been disproportionately affected by the COVID-19 pandemic [10,11,12], even though the precise magnitude and extent of such an impact are unknown.

Very few studies have explored this topic. For instance, Berman and coauthors [13] have found that the implementation of some NPIs, such as social distancing, cancelations/bans and other restrictions, can be associated with discriminatory attitudes among people living with HIV (PLWH). Authors carried out a rapid-response, cross-sectional survey recruiting 149 PLWH and were able to reveal another face of HIV-related stigma and negative psychosocial attitudes and behaviors, which have even been amplified during the COVID-19 pandemic.

Furthermore, the United States “Centers for Disease Control and Prevention” (CDC) [14,15] have reported how sexual minorities are at higher risk for contracting COVID-19 and developing complications, in that they have a higher likelihood of suffering from underlying comorbidities that represent major risk factors for COVID-19. However, the United States “COVID-19 surveillance systems” do not collect and compile data regarding patients’ self-disclosed sexual orientations and/or gender identities. To overcome this shortcoming and to fill in this knowledge gap, the CDC have extracted and analyzed data from the “Behavioral Risk Factor Surveillance System” for the period ranging from 2017 to 2019. Researchers have aimed at computing the prevalence rate among LGBTQIA2SP+ populations of daily lifestyles, habits, and health conditions that may increase the risk for contracting the infection and, more specifically, developing severe COVID-19. Approximately five percent of the survey’s participants self-identified as members of the LGBTQIA2SP+ community and, in particular, as homosexual, lesbian, or bisexual individuals, whereas very few respondents self-identified as transgender/trans-sexual or non-binary individuals. This makes it difficult to compute reliable, precise and accurate estimates related to COVID-19 risk factors among transgender/trans-sexual and non-binary subjects, which warrants the design and development of ad hoc surveys. However, based on currently available data, sexual minorities are more likely to complain of non-communicable disorders, such as asthma and chronic respiratory disease including chronic obstructive pulmonary disease (COPD), hypertension, stroke, other cardiovascular disease, and chronic kidney disease, and to report smoking.

In an interesting study, Ko and colleagues [16] carried out an online survey-based study with the aim of comparing the COVID-19-related cognitive, affective, and behavioral constructs of health beliefs between 533 sexual-minority and 1421 heterosexual individuals based in Taiwan. The authors found that participants from sexual and gender minority (SGM) populations were not aware of increased risk factors for COVID-19 among the LGBTQIA2SP+ populations and reported lower perceived susceptibility to COVID-19. Moreover, they displayed greater self-confidence levels in effectively coping and dealing with COVID-19, and exhibited significantly fewer concerns and less anxiety about COVID-19. Furthermore, in terms of health-related behaviors and the adoption of adequate preventative measures, SGM individuals were less likely to observe and maintain good indoor ventilation and disinfect their households when compared with their heterosexual counterparts. As such, sexual orientation and gender identity are major modifying factors for the “Health Belief Model” during the COVID-19 pandemic and should be incorporated within theoretical/conceptual frameworks when public health professionals and relevant stakeholders devise and implement COVID-19-related preventative interventions.

Despite this, only less than 0.2% of the COVID-19-related scholarly literature covers the effects of the pandemic on the LGBTQIA2SP+ communities, who are tremendously under-represented and see their needs still unmet. In the UK, McGowan et al. [17] conducted a systematic literature review and concluded that the lack of LGBTQIA2SP+-specific data and the paucity of related research are a significant cause for concern, given the pre-existing disparities and underlying health inequities among these specific populations. A wide array of variables, including societal and structural factors, may have contributed to the worse outcomes observed among sexual minority communities during the COVID-19 pandemic, in terms of poor mental health, general well-being and inadequate access to healthcare provision. LGBTQIA2SP+ populations have, indeed, particular health needs that should be acknowledged, and the COVID-19 pandemic has contributed, for instance, to disrupting HIV service provision [18].

The dearth of solid and robust evidence is mainly due to the lack of surveillance programs and monitoring systems that routinely collect sexual-orientation- and gender-identity-related data, which hampers the development of serious and effective public health policies [17]. From a gender perspective, epidemiological surveys have consistently shown that men are at higher risk for contracting the coronavirus infection and developing serious illness when compared with women. However, the biological/genetic basis and underlying mechanisms accounting for such discrepancies are yet to be fully elucidated. Utilizing data from transgender/trans-sexual individuals (both men or women) could help to answer research questions concerning the pathways leading to higher morbidity and mortality rates: more specifically, if these paths are due to endocrinological (sex hormone) effects or genetic (chromosomal)/epigenetic mechanisms [17]. Handling and collecting data on sexual orientation and gender identity is a good research practice that should be routinely implemented. This would enable computing the extent and the degree to which the still-ongoing COVID-19 pandemic has widened and is still widening pre-existing health inequalities [17].

Dismantling systemic and structural suppression and discrimination against LGBTQIA2SP+ communities, who are generally unheard and invisible, as well as properly addressing institutional biases and homo-bi-transphobia, prioritizing the inclusion of sexual minorities in research studies and randomized clinical trials, represents a societal onus. All this will enable achieving a two-fold goal: (i) to contribute to a more inclusive, fair and just society, and (ii) to address scholarly questions that could not be addressed with a proper solution if not properly framed and incorporated within so-called “gender medicine”.

One year after the beginning of the outbreak, several vaccine products have been approved and licensed thanks to unprecedented efforts. However, in order to be successful, an immunization campaign should reach a sufficiently large portion of the population. LGBTQIA2SP+ communities may be hard to reach, besides dealing with specific challenges, perceived barriers and obstacles that hamper equitable access to healthcare provisions. Furthermore, vaccine hesitancy poses serious global and public health risks. According to the “Strategic Advisory Group of Experts on Immunization” (SAGE) “Working Group on Vaccine Hesitancy”, vaccine hesitancy can be defined as a complex, multi-factorial, time-, space/setting-, population/community- and vaccine-specific phenomenon that results in delays in the acceptance or even in the refusal of vaccine uptake, despite the availability of vaccines [19,20].

Several determinants impacting the intention to vaccinate have been explored. However, very few studies have explored the relationship between self-declared gender identity and sexual orientation and vaccine hesitancy. Srivastav and colleagues [21] used data from the United States National Health Interview Survey, 2013–2015, and were able to identify statistically significant differences between heterosexual and LGBTQIA2SP+ populations concerning the self-reported uptake of several vaccines, including those against human papillomavirus (HPV), hepatitis A (HepA), hepatitis B (HepB), and influenza viruses. For instance, bisexual females reported higher HPV receipt rates than their heterosexual female counterparts (51.6% versus 40.2%), whereas homosexual males self-disclosed higher HepA and HepB vaccine acceptance levels when compared with their heterosexual peers (40.3% and 53.6% versus 25.4% and 32.6%, respectively). Furthermore, bisexual female subjects reported higher HepA and HepB coverage levels than heterosexual females (33.9% and 58.5% versus 23.5% and 38.4%, respectively) and higher HepB coverage than lesbian female individuals (45.4%). Interestingly, bisexual participants self-disclosed lower influenza receipt rates than homosexual/lesbian and heterosexual adults (34.1% versus 48.5% and 43.8%, respectively). With the exception of the statistically significant associations between self-disclosed sexual orientation and/or gender identity and greater likelihood of uptake of HPV, HepA, HepB, and influenza vaccines, remaining data did not support the hypothesis of a correlation between a specific sexual orientation/gender identity and the intention/willingness to vaccinate. This held true, when adjusting for confounding factors, in that health status or other behavioral features had no statistically consistent and robust relationships with vaccination uptake among all the populations under study.

In another interesting study conducted in the United States, Adjei Boakye et al. [22] found that, even though lesbian and bisexual women had a higher likelihood of HPV vaccine initiation and completion when compared with their heterosexual counterparts, the HPV vaccine uptake in this specific population significantly failed to achieve the “Healthy People 2020” target.

Specifically concerning sexual orientation and/or gender identity as a key determinant/predictor of the intention to vaccinate, there are scarce and conflicting results in the literature. In more detail, there is a dearth of data concerning vaccine acceptance for other vaccine products besides vaccines against HPV and influenza, and, as previously mentioned, vaccine hesitancy is vaccine-specific. Therefore, there is a strong need to routinely collect sexual-orientation and/or gender-identity data, while, of course, preserving privacy and ensuring compliance with legal and ethical standards.

In the United States, Stephenson and coworkers [23] sampled from the “Love and Sex in the Time of COVID-19” study, in the period from November 2020 to January 2021, that is to say, before the start of the vaccine rollout program. The authors investigated “COVID-19 pandemic optimism”, “vaccine optimism” and the intention/willingness to vaccinate among 290 gay, bisexual, and other men who have sex with men (GBMSM) populations. More specifically, the authors quantitatively assessed the determinants of COVID-19 vaccine uptake in terms of three dimensions of a psychometric construct related to vaccine beliefs (namely, (i) the perception of the likelihood of a COVID-19 vaccine soon becoming available, (ii) the perception of when a COVID-19 vaccine would become available (for example, within 6 months), and, finally, (iii) the likelihood of accepting a COVID-19 vaccine). Differently from other studies, including the previous study by Srivastav et al. [21], ethnic (Black/African American) and sexual minority (GBMSM living with HIV) communities had higher levels of COVID-19 pandemic and vaccine optimism and were more likely to be willing to take a COVID-19 vaccine. Male participants who perceived a higher prevalence rate of COVID-19 among their peers and social networks, including friends and sex partners, and those who had decreased their sexual activities or modified their sexual habit patterns (i.e., by reducing their sex partners), were more likely to be willing to be vaccinated against COVID-19. Only approximately 14% of the participants were skeptical about the end of the COVID-19 pandemic and were unwilling to take a COVID-19 vaccine [23].

In another study conducted in the United States, Teixeira da Silva et al. [24] carried out an online cross-sectional study focused on the intention to adopt a HIV biomedical prevention technology targeting specific populations and communities at higher risk for HIV sero-conversion. The study population consisted of a sample of 1350 predominantly homosexual (61.6%), Black/Afro-American (57.9%), cis-gender (95.7%) males with an average age of 32.9 years. Lower levels of COVID-19 vaccine acceptance characterized participants displaying high levels of medical mistrust and societal concerns regarding COVID-19 immunization stigma and exhibiting low levels of altruism and generosity. Ethnicity was a significant determinant, impacting the choice to vaccinate, with Black/Afro-American participants being less willing to take a COVID-19 vaccine, and Asian subjects being more accepting, when compared with their White counterparts.

As of 26 September 2021, more than 6.11 billion COVID-19 vaccine doses had been administered globally, equal to 80 doses for every 100 people. However, the implementation of the COVID-19 vaccine rollout strategies and programs is characterized by a high amount of heterogeneity among countries and territories, with several health disparities and inequities. The percentage of fully vaccinated people widely ranges from 84% (in the United Arab Emirates) to less than 0.1% (in Congo).

Public health organizations and authorities should have the onus to ensure an equitable implementation of the COVID-19 vaccine rollout strategies and prevent the widening of health disparities, which further delay global economic and clinical recovery. Specifically concerning SGM populations, as stated by Stephenson et al. [23], to reach the levels of vaccination necessary to effectively control the pandemic and to achieve herd immunity, it is of crucial importance to better understand the characteristics of those experiencing vaccine hesitancy and then tailor and customize public health messages to their set of unique perceived barriers, structural obstacles and motivations.

In their study, Garg and coworkers [25] performed a systematic review of the literature. The authors found that there exist several studies that have investigated the factors and determinants underlying vaccine hesitancy in different contexts and settings, even though members of the LGBTQIA2SP+ populations remain tremendously under-represented or even misrepresented in a large portion of these investigations. The COVID-19 vaccine acceptance among non-binary individuals ranged from 28.90% to 56.25% [25,26,27]. Moreover, based on the very few studies performed specifically focusing on the LGBTQIA2SP+ populations, including the seminal study by Teixeira da Silva et al. [24], the authors could identify some predictors including (i) concerns and worries about vaccine safety and the insurgence of potential side effects, (ii) vaccine efficacy/effectiveness, and (iii) a history of bad experiences with healthcare providers, such as physicians, practitioners and other allied health professionals.

Summarizing what we have learnt from the existing scholarly literature and, in particular, from the study by Garg and coauthors [25], (i) the COVID-19 pandemic, displaying a syndemic nature, has disproportionately affected the LGBTQIA2SP+ populations, who (ii) have a higher likelihood of reporting major risk factors for contracting the virus and developing complications. (iii) However, accurately computing the impact imposed by COVID-19 among the LGBTQIA2SP+ communities is hindered by the lack of specific data. (iv) Sexual orientation and/or gender identity appear to impact the intention to vaccinate against COVID-19, and (v) sexual minorities appear to be reluctant to take a COVID-19 vaccine, iv) even though the vaccine hesitancy rate considerably varies among the very few existing studies.

It is essential for global and public health workers to (i) acknowledge LGBTQIA2SP+ communities, give them a voice, and listen to their particular healthcare needs, and (ii) empower the LGBTQIA2SP+ populations and increase their resilience, enhancing their health literacy, by explaining, for example, that being on pre-exposure prophylaxis (PrEP) for HIV does not represent a contraindication for the uptake of a COVID-19 vaccine. Moreover, all the relevant stakeholders, including governmental bodies and authorities, public health decision makers, and policymakers, as well as healthcare providers, should make their best efforts to rebuild the trust of the LGBTQIA2SP+ communities about immunization practices. Members of sexual minorities should be involved in randomized clinical trials and other epidemiological observational studies and surveys.

Multi-component interventional programs aimed at counteracting health disparities and iniquities, building confidence, and devising and implementing ad hoc public health policies tailored to the needs of the LGBTQIA2SP+ communities should be an effective tool with which to include this specific population and to fight the COVID-19 pandemic.

As the Section Editor-in-Chief of “Immunology and Vaccines”, a section of *Infectious Disease Reports*, and as an out-and-proud member of the LGBTQIA2SP+ community, I am twice “proud” to introduce to the readers of this journal an important article [25] that addresses a very timely and urgent topic (COVID-19 vaccine hesitancy among sexual-minority community members) in an evidence-based fashion. Moreover, this editorial calls for more research in the field to fill in the existing gaps in knowledge and to expand our understanding of a pandemic from a syndemic and gender medicine perspective.

## Data Availability

Not applicable.
